# GPR30 regulates the EGFR-Akt cascade and predicts lower survival in patients with ovarian cancer

**DOI:** 10.1186/1757-2215-5-35

**Published:** 2012-11-19

**Authors:** Satoe Fujiwara, Yoshito Terai, Hiroshi Kawaguchi, Masaaki Takai, Saha Yoo, Yoshimichi Tanaka, Tomohito Tanaka, Satoshi Tsunetoh, Hiroshi Sasaki, Masanori Kanemura, Akiko Tanabe, Yoshiki Yamashita, Masahide Ohmichi

**Affiliations:** 1 Department of Obstetrics and Gynecology, Osaka Medical College, 2-7, Daigaku-machi, Takatsuki, Osaka, 569-8686, Japan

**Keywords:** G protein-coupled receptor 30, GPR30, 7-transmembrane estrogen receptor, EGFR, Akt, ERα; Clear cell carcinomas, Prognostic factor

## Abstract

**Objectives:**

G protein-coupled receptor 30 (GPR30) is a 7-transmembrane estrogen receptor that functions alongside traditional estrogen receptors to regulate the cellular responses to estrogen. Recent studies suggest that GPR30 expression is associated with a poor prognosis, and that this is due to the GPR30-mediated transactivation of the EGFR in breast cancer. However, the biological contribution of GPR30 in ovarian cancer remains unclear. The purpose of this study was to elucidate the relationships between GPR30 expression and the clinicopathological findings, and to determine how the signaling cascade influences the prognosis of ovarian cancer.

**Methods:**

The expression levels of GPR30, EGFR, ERα, and ERβ were analyzed using an immunohistochemical analysis, and their correlations with the clinicopathological features were examined in 10 patients with borderline malignant tumors and 152 patients with epithelial ovarian cancer. We also examined whether GPR30 signaling activates the EGFR-Akt pathway in an ovarian cancer cell line (Caov-3) by a Western blotting analysis.

**Results:**

The GPR30 expression in ovarian carcinomas was significantly higher than that in borderline malignancies (p=0.0016), and was not associated with the expression of the EGFR, ERα, or ERβ. The expression of GPR30 in clear cell carcinomas was significantly lower than that in other subtypes of cancer (P <; 0.001). The expression of both GPR30 and EGFR was significantly associated with a poor prognosis in terms of the progression-free survival rate. The phosphorylation of the EGFR and Akt could be significantly enhanced by G1 (p <; 0.05) and inhibited by a Src family kinase inhibitor.

**Conclusion:**

The expression of both GPR30 and EGFR is associated with a poor outcome in ovarian cancer, and GPR30 increases the phosphorylation of Akt via the EGFR in ovarian cancer cells. The regulation of GPR30 might be a potentially useful new therapeutic target in ovarian cancer.

## Introduction

Ovarian cancer is the most common cause of gynecological cancer-related death. Approximately 70% of all patients with ovarian cancer are diagnosed at an advanced stage, and 60% to 80% of patients die of the disease [1]. The main reasons for the poor prognosis are the high recurrence rate and resistance to second-line chemotherapeutics. Therefore, the development of new therapies is critical for the treatment of ovarian cancer patients.

Estrogens are major regulators of growth and differentiation in the normal ovaries, and also play an important role in the progression of ovarian cancer. Likewise, a marked proliferative response to estrogens was shown in ovarian surface epithelial cells, which are the site of 90% of malignancies [2], and an increased risk of ovarian tumors was observed in postmenopausal patients receiving estrogen replacement therapy [3-5]. The biological effects of estrogens are classically mediated by the estrogen receptors (ER)α, and ERβ, which function as hormone-inducible transcription factors that bind to the estrogen-responsive elements (EREs) located within the promoter regions of target genes [6]. Moreover, it has been suggested that the nongenomic actions of estrogens, as well as the genomic effects, are susceptible to interference from environment estrogens [7]. However, the precise identity and function of many steroid membrane receptors are still controversial in terms of their specific molecular interactions with endogenous and environmental estrogens. Many studies have demonstrated that ERβ is highly represented in normal ovarian epithelial cells and benign tumors, whereas ERα is the main form expressed in ovarian cancer [8-11]. The ERβ mRNA expression is inversely correlated with tumor progression, while the ERα mRNA expression is positively correlated with the progression [10,12]. The ER α/β mRNA ratio is markedly increased in ovarian cancer [9]. In contrast with breast cancer, the prognostic value of the ERα or ERβ status, as well as the prediction of the responsiveness to anti-estrogen treatment, have not been clearly established for ovarian cancer [3-15].

The G protein-coupled receptor 30 (GPR30), which mediates the nongenomic signaling of 17-beta-estradiol (E2), is widely expressed in cancer cell lines and primary malignant tumors of the breast, endometrium, prostate and lungs [16,17], and is strongly associated with the proliferation, invasion, metastasis, and drug resistance of various cancer cell lines [18-24]. GPR30 protein expression correlates with the clinical and pathological biomarkers of a poor outcome in breast cancer and endometrial cancer [25,26]. Recent studies have shown that GPR30 exerts its effects through the activation of the epidermal growth factor receptor (EGFR) transduction pathway in endometrial cancer, breast cancer, and thyroid cancer cells [19,20,27-29]. Moreover, Filardo et al. found that estrogen rapidly activated extracellular signal-related kinase (ERK) 1/2 via EGFR transactivation in breast cancer cell lines, regardless of their ER status [20]. Signaling via the EGFR leads to multiple downstream events; for example, activation of phospholipase C (PLC), the phosphatidylinositol-3 kinase (PI3K)/Akt pathway and MAPK. The EGFR is reported to be present in 33-75% [30] of ovarian cancers and has been implicated in both the growth and progression of this disease [31]. Akt activation is closely related to cancer cell growth, because it affects cancer cell survival, proliferation (leading to an increased cell number) and growth (increasing the cell size) [32-34].

Only one previous study has shown that high levels of GPR30 expression predict a poor prognosis in ovarian cancer [35]. This is not yet sufficient evidence of a role of GPR30 in ovarian cancer. Therefore, it was necessary to clarify whether GPR30 and the GPR30-dependent activation of MAPK-ERK1/2 via EGFR transactivation is important in ovarian cancer. In the current study, we evaluated the relationship between GPR30 expression and patient clinicopathological factors by immunohistochemistry in ovarian cancer specimens, and evaluated whether GPR30 mediates Akt activation via the EGFR, leading to a poor prognosis for ovarian cancer patients.

## Materials and methods

### Patients

This study was reviewed and approved by the Institutional Review Board of the Osaka Medical College and informed consent was obtained from all patients. The study included 162 patients with either primary epithelial ovarian cancer (152) or low malignant potential tumors [10] who underwent a diagnosis and surgical resection in the Department of Gynecology of Osaka Medical College Hospital in Japan between 2001 and 2009. In the 162 epithelial ovarian cancer and borderline malignant patients, an effort was made to perform optimal surgical cytoreduction and adequate staging, which included at least a total abdominal hysterectomy with bilateral salpingo-oophorectomy, omentectomy, peritoneal washings and retroperitoneal lymphadenectomy. The histology of all tumors was determined by a gynecological pathologist according to the WHO criteria (World Health Organization).

### Immunohistochemistry

Five-micron sections from tumor tissues, which were formalin-fixed and embedded in paraffin, were prepared for the immunohistochemical analyses. The expression of GPR30 was analyzed as follows: Tumor sections were incubated at 4°C for 18 h with a GPR30-specific antibody, a rabbit polyclonal affinity-purified antibody directed against the C-terminal of GPR30, at a 1:50 dilution (LifeSpan BioSciences, Inc.). The EGFR expression was analyzed using the EGFR Pharm Dx kit (Dako Cytomation). The expression levels of ERα and ERβ were analyzed using a Dako Cytomation ER Pharm assay.

The interpretation of the immunohistochemical staining results was performed by two independent gynecological oncologists (who were accustomed to diagnosing gynecological malignancies) who were blinded to the clinicopathological data. For each core, the staining intensity (graded considering 0 as negative, 1+ as weak, 2+ as moderate, and 3+ as strong) and the percentage of cells staining positive (0 – 100%) were determined. The overexpression of GPR30 and the EGFR was defined to exist if 50% or more of the tumor cells exhibited cytoplasmic or membranous staining with a staining intensity of 2+ or more. The overexpression of ERα and ERβ was defined to exist if 1% or more of the tumor cells exhibited nuclear staining with a staining intensity of 1+ or more.

### Cell culture

One human ovarian mucinous adenocarcinoma cancer cell line, Caov-3, which was obtained from the American Type Culture Collection (Rockville, MD, USA), was grown in phenol red free DMEM containing 10% dextran-coated, charcoal-treated fetal calf serum, 100 units/ml penicillin, and 100 μg/ml streptomycin in a humidified atmosphere of 5% CO_2_ with 95% air at 37°C.

### Expression plasmids and cDNA transfection

To make the pcDNA3.1-GPR30 expression construct, the cDNA of the full length GPR30 was amplified by PCR using a human mammary gland cDNA library as the template. For the transfection of each sample, oligomer-Lipofectamine plus complexes were prepared as follows: 100 pmol of cDNA oligomer were diluted in 250 μl of Opti-MEM (Invitrogen). The Lipofectamine plus was mixed gently before use, and then a 5 μl aliquot was diluted in 250 μl of Opti-MEM, mixed gently, and incubated for 5 min at room temperature. After the 5 min incubation, the diluted oligomer was combined with the diluted Lipofectamine plus, mixed gently, and incubated for another 20 min at room temperature. The oligomer-Lipofectamine plus complexes were added to each well containing cells and medium, and mixed gently by rocking the plate back and forth. The cells were incubated at 37°C in a CO_2_ incubator for 24 h, then the cells were prepared for each assay.

### Proliferation assay

After the seeding, the test cells were incubated with phenol red free DMEM containing 10% dextran-coated, charcoal-treated fetal calf serum for 24 hours in 96 well plates. The changes in cell proliferation were examined by the addition of G1, which is a selective agonist of GPR30, or G15, which is a selective antagonist, with serum free DMEM for 48 hours. The number of Caov-3 cells after 48 hours of stimulation was determined by measuring the dissolved formazan products after the addition of MTS. All experiments were carried out in quadruplicate, and the cell viability was expressed as the ratio of the number of viable cells with G1 stimulation to that of cells without stimulation.

### Western blot analysis

The cells were serum-starved and stimulated with PBS (phosphate-buffered saline) or 100 nM G1 for 5 min or 15 min. Cells were then washed twice in ice-cold PBS and lysed, and the cytoplasmic and nuclear fractions were separated using a Nuclear Extract Kit (Active Motif, Carlsbad, CA, USA). To detect all of the proteins, equal amounts of cytoplasmic proteins were separated by SDS-polyacrylamide gel electrophoresis and transferred to nitrocellulose membranes. Blocking was done in 10% bovine serum albumin in 1x Tris-buffered saline. The Western blot analyses were performed with various specific antibodies. The immunoreactive bands in the immunoblots were visualized with horseradish peroxidase-coupled goat anti-rabbit immunoglobulin using an enhanced chemiluminescence Western blotting system (ECL Plus, GE healthcare Life Sciences, Pittsburgh, PA, USA). All Western blots were checked for equal protein loading using Ponceau staining.

### Statistical analysis

The statistical analyses in this study were carried out with the Stat View statistical software package (SAS Institute, Cary, NC, USA). Fisher’s exact probably test was used to evaluate the correlations between the immunohistochemical and clinical data. The endpoints investigated were the progression-free survival and overall survival. The progression-free survival was defined as the time from the first day of chemotherapy until disease progression (based on the findings of imaging studies). Overall survival was defined as the time from the first day of chemotherapy to death from any cause. The univariate and multivariate analyses of the histology, progression-free survival and overall survival were determined with the Kaplan-Meier method using the log-rank test and the Cox proportional hazards model, respectively. Differences with p-values <; 0.05 were considered to be statistically significant.

## Results

### Correlations of GPR30, EGFR, ERα and ERβ with the clinicopathological features of ovarian carcinomas

**Figure 1:**
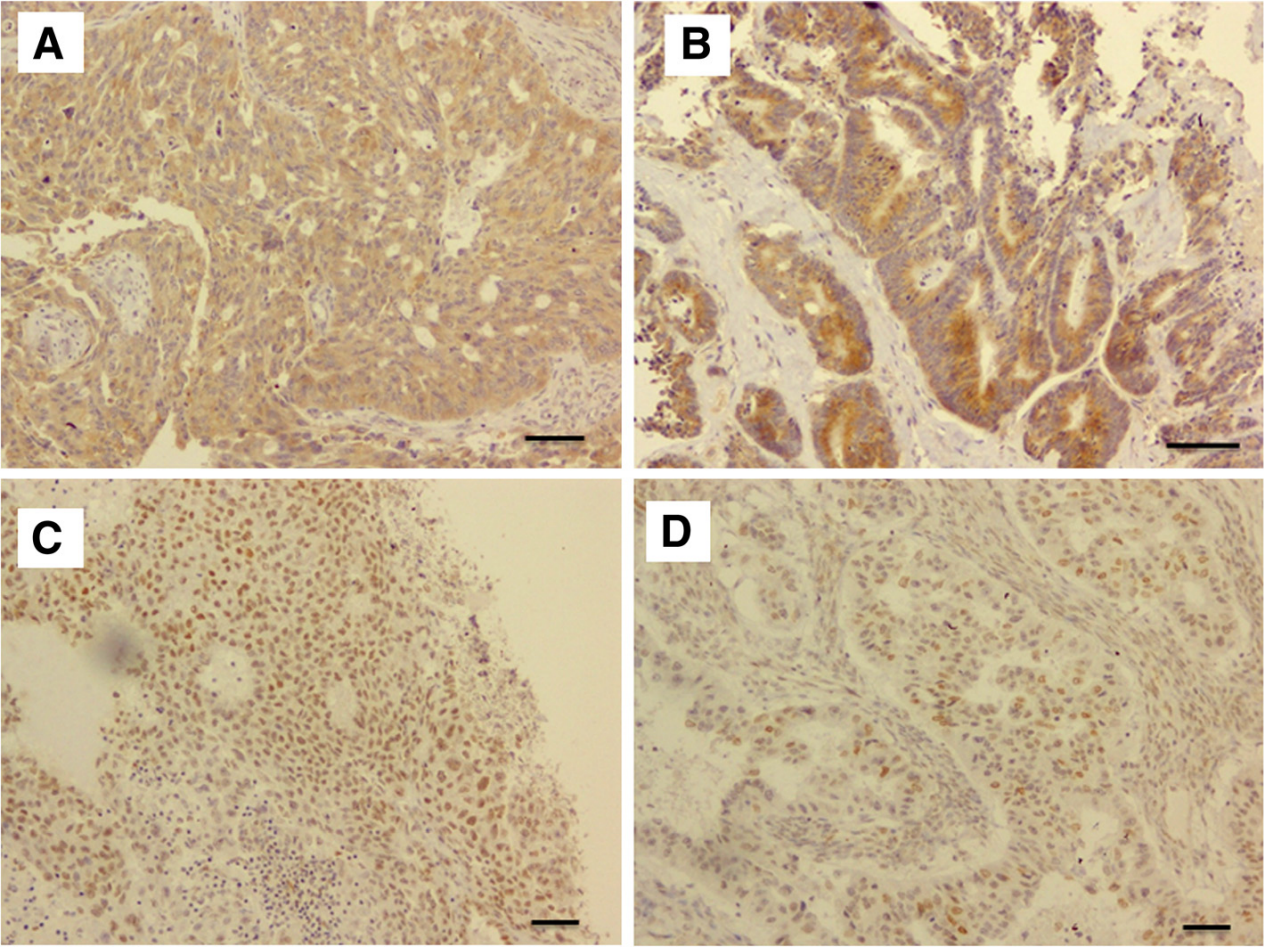
**Representative examples of immunohistochemically-stained sections.** Representative examples of immunohistochemically-stained sections from tumor specimens that were positive for GPR30 (**A**), EGFR (**B**), ERα (**C**) and ERβ (**D**). Scale bars represent 100 μm.

The clinical and pathological data for the patients are shown in Table 1. Of the 162 investigated patients, 10, 39, 14, 77 and 22 were categorized to have a borderline malignancy, and stage I, II, III and IV ovarian cancer, respectively. Of the 162 cases, 10 were borderline malignant tumors, 61 were histologically diagnosed to be serous adenocarcinoma, 30 endometrioid adenocarcinoma, 19 mucinous adenocarcinoma and 29 were diagnosed to be clear cell adenocarcinoma. In the tumor specimens obtained by surgical resection, the expression levels of the GPR30, EGFR, ERα and ERβ proteins were evaluated. A representative example of the immunostaining analysis is shown in Figure 1. The expression of GPR30 and EGFR was detected mainly in the cytoplasm, and the ERα and ERβ expression was detected mainly in the nuclei of the tumor specimens.

**Table 1:** Characteristics of borderline malignancy and epithelial ovarian cancer cases

**Variables**	**Number of patients (%)**
	N=162
Age (years)	54.1 ± 12.5
Postmenopausal	96
Premenopausal	66
BMI	22.0 ± 3.4
Histology Borderline malignancy	10 (6.1%)
Serous adenocarcinoma	61 (37.6%)
Endometrioid adenocarcinoma	30 (18.5%)
Mucinous adenocarcinoma	19 (11.7%)
Clear cell adenocarcinoma	29 (17.9%)
Other	13 (8.0%)
FIGO stage I	49 (30.2%)
II	14 (8.6%)
III	77 (47.5%)
IV	22 (13.5%)

We also examined the relationships between the clinicopathological factors and the immunohistochemical staining patterns, the results of which are shown in Tables 2 and 3. There were no significant associations between the GPR30 expression and the patient age or menstrual status. However, the GPR30 expression correlated with the mean body mass index (BMI in kg/cm^2^) (p<;0.04). The GPR30 expression in ovarian carcinomas was significantly higher than that in borderline malignancies (p=0.0016). Interestingly, the GPR30 expression was significantly related to the histological subtype (p<;0.001). GPR30 expression was observed in 75.4% (46/61) of serous adenocarcinomas, 83.3% (25/30) of endometrioid adenocarcinomas and 73.7% (14/19) of mucinous adenocarcinomas, while 20.7% (6/29) of clear cell carcinomas were observed to express the protein.

**Table 2:** Correlations between GPR30 and EGFR expression and clinicopathological factors

**Variables**	**Expression of GPR30**		**p value**	**Expression of EGFR**		**p value**
	**Positive**	**Negative**		**Positive**	**Negative**	
Age	54.0 ± 12.7	54.3 ± 12.2	0.49	54.3 ±11.9	53.9 ±13.5	0.84
Premenopausal	40 (60.6%)	26 (39.4%)	0.80	27 (40.9%)	39 (59.1%)	0.39
Postmenopausal	60 (60.6%)	36 (39.4%)		33 (33.3%)	63 (66.7%)	
BMI	21.5 ± 2.7	22.6 ± 4.2	0.04	21.4±3.6	22.2±2.8	0.11
Borderline malignancy	1 (10.0%)	9 (90.0%)	0.0016	0 (0%)	10 (100%)	0.012
FIGO stage I	26 (66.7%)	13 (33.3%)	0.31	13 (33.3%)	26 (66.7%)	0.77
II	11 ( 78.6%)	3 (21.4%)		7 (50.0 %)	7 (50.0%)	
III	47 (61.3%)	30 (38.7%)		32 (41.6%)	45 (58.4%)	
IV	15 (68.2%)	7 (31.8%)		8 (36.4%)	14 (63.6%)	
Histology						
Borderline malignancy	1 (10.0%)	9 (90.0%)	0.0016	0 (0%)	10 (100%)	0.012
Serous adenocarcinoma	46 (75.4%)	15 (24.6%)	<;0.001	24 (39.3%)	37 (60.7%)	0.42
Endometrioid adenocarcinoma	25 (83.3%)	5 (16.7%)		11 (36.7%)	19 (63.3%)	
Mucinous adenocarcinoma	14 (73.7%)	5 (26.3%)		8 (42.1%)	11 (57.9%)	
Clear cell adenocarcinoma	6 (20.7%)	23 (79.3%)		14 (48.3%)	15 (51.7%)	
Others	8 (61.5%)	5 (38.5%)		3 (23.1%)	10 (76.9%)	
Recurrence			0.30			0.31
≤ 6M	13 (68.4%)	6 (31.6%)		9 (47.4%)	10 (52.6%)	
> 6M	43 (67.2%)	21 (32.8%)		20 (31.3%)	44 (68.7%)	
No recurrence	44 (55.7%)	35 (44.3%)		31 (39.2%)	48 (60.8%)	
5-year survival			0.56			0.86
Alive	63 (61.8%)	39 (38.2%)		35 (34.3%)	67 (65.7%)	
Dead	37 (61.7%)	23 (38.3%)		25 (41.7%)	35 (58.3%)	

**Table 3:** Correlations between the clinicopathological factors and estrogen receptors (ERa, ERb)

**Variables**	**Expression of ERα**		**p value**	**Expression of ERβ**		**p value**
	**Positive**	**Negative**		**Positive**	**Negative**	
Age	55.9 ±13.1	52.6±13.1	0.12	55.8 ±12.3	53.6±11.9	0.37
Premenopausal	24 (36.4%)	42 (63.6%)	0.24	16 (24.2%)	50 (75.8%)	0.85
Postmenopausal	41 (42.7%)	55 (57.3%)		20 (20.8%)	76 (79.2%)	
BMI	22.3 ±2.5	21.7 ±2.6	0.24	21.7 ± 4.5	22.0 ± 2.9	0.75
Borderline malignancy	5 (50.0%)	5 (50.0%)	0.72	6 (60.0%)	4 (40.0%)	0.08
FIGO stage I	10 (25.6%)	29 (74.4%)	0.99	14 (35.9%)	25 (64.1%)	0.06
II	9 (64.3%)	5 (35.7%)		2 (14.3 %)	12 (85.7%)	
III	34 (44.2%)	43 (55.8%)		14 (18.2%)	63 (81.8%)	
IV	7 (31.8%)	15 (68.2%)		0 (0%)	22 (100%)	
Histology						
Borderline malignancy	5 (50.0%)	5 (50.0%)	0.72	6 (60.0%)	4 (40.0%)	0.08
Serous adenocarcinoma	32 (52.5%)	29 (47.5%)	0.004	11 (18.0%)	50 (82.0%)	0.14
Endometrioid adenocarcinoma	16 (53.3%)	14 (46.7%)		4 (13.8%)	26 (86.2%)	
Mucinous adenocarcinoma	5 (26.3%)	14 (73.7%)		8 (42.1%)	11 (57.9%)	
Clear cell adenocarcinoma	3 (10.3%)	26 (89.7%)		6 (20.7%)	23 (79.3%)	
Others	4 (30.8%)	9 (69.2%)		1 (7.7%)	12 (92.3%)	
Recurrence			0.14			0.06
≤ 6M	8 (42.1%)	11 (57.9%)		4 (21.1%)	15 (78.9%)	
> 6M	31 (48.4%)	33 (51.6%)		10 (15.6%)	54 (84.4%)	
No recurrence	26 (32.9%)	53 (67.1%)		22 (27.8%)	57 (72.2%)	
5-year survival			0.47			0.06
Alive	41 (40.2%)	61 (59.8%)		26 (25.4%)	76 (74.6%)	
Dead	24 (40.0%)	36 (60.0%)		10 (16.7%)	50 (83.3%)	

No significant associations between the EGFR, ERα and ERβ expression and the patient age, menstrual status or BMI were observed. The EGFR expression level in ovarian carcinomas was significantly higher than that in borderline malignancies (p=0.012), although the ERα and ERβ expression in ovarian carcinomas was not significantly different from that in borderline malignancies. Interestingly, the ERα expression levels were also significantly related to the histological subtype (p=0.004).

### Prognostic impact of GPR30, EGFR, ERα and ERβ expression in ovarian carcinoma

**Figure 2:**
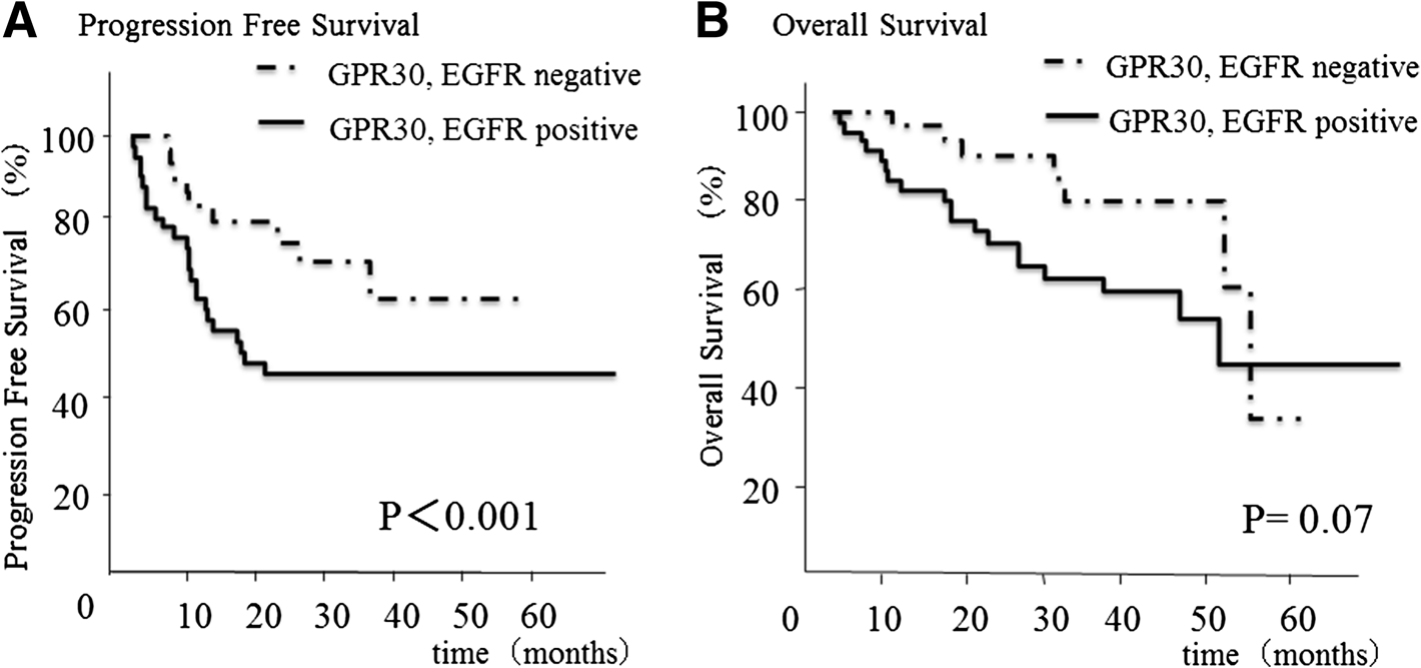
**Correlation of the co-expression of GPR30 and EGFR with the progression-free survival or overall survival.** (**A**) A high level of tumor co-expression of GPR30 and EGFR was significantly associated with a poorer progression-free survival (p<;0.001). (**B**) There was no significant relationship, but there was a tendency for there to be a correlation between the overall survival and the co-expression of GPR30 and EGFR (P=0.07).

We also examined the relationship between the staining intensity of various markers and patient survival. The median survival time for all patients was 5.2 years. Recent studies have shown that GPR30 is expressed in a variety of estrogen-responsive cancer cells, and that it activates the epidermal growth factor receptor (EGFR) transduction pathway in various malignancies [19,20,27-29]. We therefore examined the GPR30-related status, as represented by the co-expression of GPR30 and the EGFR, and its relationship to the patients’ survival. A high tumor co-expression of GPR30 and EGFR was significantly associated with a poorer progression-free survival (p<;0.001) (Figure 2). However, the individual GPR30 and EGFR expression levels, and the ERα and ERβ expression levels, were not significantly related to the survival. 

### G1 stimulates the proliferation of Caov-3 ovarian carcinoma cells

**Figure 3:**
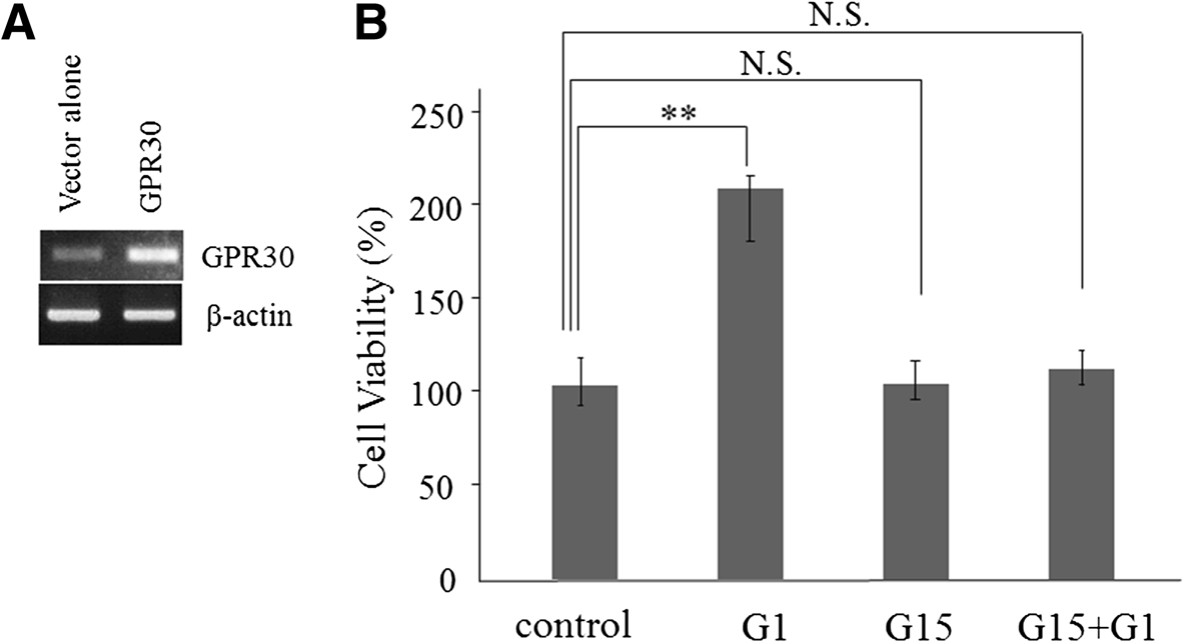
**The proliferation of the Caov-3 cells following stimulation with G1, a selective GPR30 agonist, and G15, a selective GPR30 antagonist.** Caov-3 cells were transfected with the pcDNA3.1 vector (Vector alone) or pcDNA3.1-GPR30 (GPR30). (**A**) The mRNA expression of GPR30 in Caov-3 cells was confirmed by semiquantitative RT-PCR. (**B**) The proliferation of the Ca-ov3 cells transfected with pcDNA3.1-GPR30 was examined using the MTS assay. The Caov-3 cells transfected with pcDNA3.1-GPR30 were treated with G1 (100 nM), G15 (100 nM), or both G1 and G15 (100 nM each) for 48 hours. The cell number is expressed as a percentage of the control (100%) which Caov-3 cells transfected with pcDNA3.1-GPR30. The values shown represent the means ±SE of three independent experiments performed in quadruplicate in three different passages of the cell lines. Significant differences are shown by asterisks: **, *p*<;0.01.

In recent studies, GPR30 was reported to regulate the GPR30-dependent activation of MAPK-ERK1/2 via EGFR transactivation in breast cancer and endometrial cancer [17,20]. However, it is unclear whether GPR30-mediated Akt activation via EGFR transactivation occurs in ovarian cancer. To assess this possibility, we first evaluated the ability of G1, a selective GPR30 agonist, to activate a transiently transfected GPR30 gene in an ovarian cancer cell line (Caov-3 cells). The mRNA expression of GPR30 in Caov-3 cells was evaluated by semi quantitative RT-PCR (Figure 3A). The mRNA expression of GPR30 in Caov-3 cells that were not transfected with the GPR30 gene was significantly lower than that in Caov-3 cells transfected with the GPR30 gene (Figure 3A). The proliferation of the Caov-3 cells was examined using the MTS assay. As shown in Figure 3, a concentration of 100 nM of G1 enhanced the proliferation of Caov-3 cells for 48 h in culture ((p=0.0016), and 100 nM of G15, which is a selective GPR30 antagonist, inhibited the G1-induced proliferation of the Caov-3 cells (Figure 3B) (Western blotting analysis; Additional file 1: Figure S3). 

### GPR30 signaling initiates EGFR-Akt signaling in ovarian cancer cells

**Figure 4:**
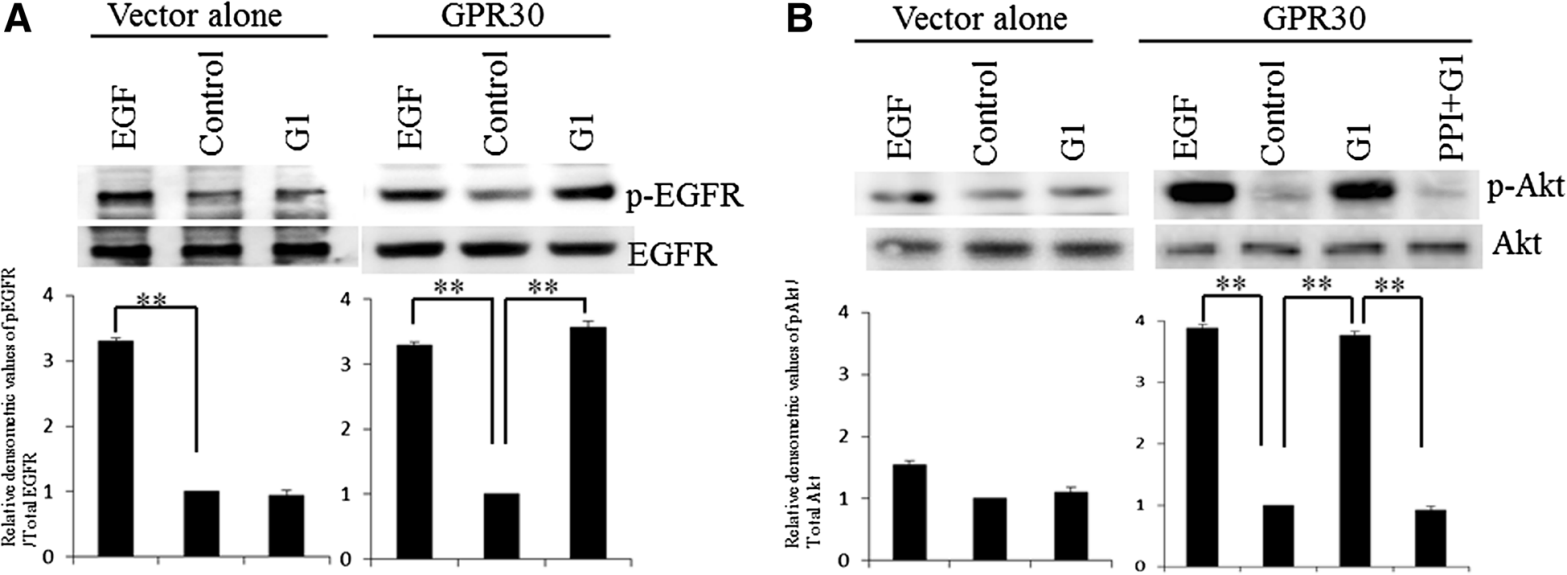
**G1, a selective agonist of GPR30, induced the phosphorylation of the EGFR and Akt.** (**A**) After treatment with or without 10 nM of EGF for 5 min or 100 nM of G1 for 5 min, cells were harvested and used to prepare cell lysates. The lysates were subjected to SDS-PAGE and blotted with anti-phospho EGFR (upper panel) or anti-EGFR (lower panel) antibodies. (**B**) Cells were treated with or without 10 nM of EGF for 5 min, 100 nM of G1 for 5 min, or 10 nM of PP1, followed by G1 treatment. Cell lysates were subjected to SDS-PAGE and blotted with anti-phospho Akt (upper panel) or anti-Akt (lower panel) antibodies. The values shown represent the means ± standard deviation from at least three separate experiments. Significant differences are indicated by asterisks. **; p<;0.01 (Additional file 2: Supplemental data 1).

Next, we ascertained that in Caov-3 cells, rapid phosphorylation of the EGFR and Akt were induced by G1 via Src using a Western blot analysis. The mRNA expression of GPR30 in the Caov-3 cells was confirmed by semiquantitative RT-PCR (Figure 3A). As shown in Figure 4A, the phosphorylation of the EGFR could be significantly enhanced by the addition of 100 nM G1 (p<;0.01), to a similar extant as was observed using EGF. The phosphorylation of Akt could also be significantly enhanced by 100 nM of G1 (p<;0.01) or EGF, and could be inhibited by PP1, which is a Src family kinase inhibitor (Figure 4B, A2780 cells; Additional file 2: Figure S1, RMG-1 cells; Additional file 3: Figure S2).

## Discussion

The current study revealed that the novel estrogen-responsive receptor, GPR30, is preferentially active in ovarian cancer, similar to breast, endometrial, prostate and lung carcinomas. Moreover, we also showed that GPR30 was expressed at higher levels and was more frequently observed in ovarian cancer than in borderline malignant tumors. These data are consistent with a previous report [35].

Estrogens stimulate the proliferation of ovarian cancer cell lines and normal ovarian surface epithelial cells in culture [36,37]. However, the ER is present in only ~60% of ovarian cancers [13]. We demonstrated that GPR30 overexpression was not associated with the ER expression. These data suggest the presence of a complicated relationship between GPR30 and the ER. Although GPR30 is widely expressed in cancer cell lines and primary malignant tumors of the breast, endometrium, prostate and lungs [16,17], the role of GPR30 in ovarian carcinoma was unclear. Albanito et al. reported that GPR30 was involved in the proliferation of ovarian cancer cells [38]. The signaling pathways employed by GPR30 activation have not yet been fully elucidated. Figaro et al. showed that GPR30 regulated the activation of MAPK-ERK1/2 via EGFR transactivation in ER-negative breast cancer cell lines [20]. This suggests that GPR30 is the sole receptor responsive to estrogen that leads to EGFR transactivation in ER-negative breast cancer. The EGFR is reported to be present in 33-75% [30] of ovarian cancers, and has been implicated in both the growth and progression of this disease [31]. In the present study, we proved that G1, a selective GPR30 agonist, induces the proliferation of Caov-3 ovarian cancer cells, and that G15, a selective GPR30 antagonist, inhibits the G1-induced proliferation of Caov-3 cells. Moreover, G1, like EGF, significantly enhanced the activation of both the EGFR and Akt signaling pathways, and the activation of these pathways was inhibited by PP1, a Src family kinase inhibitor. This might suggest that GPR30 is involved in a signaling cascade that is transduced via the EGFR, which leads to a poor prognosis for ovarian cancer, since the phosphorylation of Akt via the EGFR is key to the development and/or progression of ovarian cancer [39].

In the present study, we demonstrated that the co-expression of GPR30 and the EGFR was associated with a poorer progression-free survival in patients with ovarian cancer, although we could not confirm the presence of a correlation between GPR30 and the survival of ovarian cancer patients, as was the case in a previous report [40]. Our clinical results support the idea that GPR30 regulates an EGFR cascade, which is closely related to cancer cell growth and the survival of ovarian cancer patients. We have previously reported that the phosphorylation of Akt leads to increased cell survival and is associated with platinum resistance due to its anti-apoptotic effects in ovarian cancer cells [34], and that it was associated with a poor overall survival in ovarian carcinoma patients [33]. In the current study, we also revealed that the cell proliferation in ovarian cancer were dependent on GPR30.

Of note, we also showed that the expression of GPR30 in clear cell carcinoma was significantly lower than that in other types of ovarian cancer. Clear cell adenocarcinoma is well known to have the worst prognosis of the various subtypes of ovarian cancer because of its resistance to chemotherapy, which has been attributed to a slow cell cycle [41]. Clear cell ovarian tumors do not express estrogen or progesterone receptors, and endometriosis that transforms into clear-cell ovarian cancer can become hormone independent during the transformation process [42]. Pandeet al. reported that GPR30 signaling induces proliferation and promotes cell cycle progression [23]. Our current results showed that there was low expression of not only ERα, but also GPR30, in clear cell adenocarcinoma, which means that clear cell adenocarcinoma is likely to have slow proliferation or slow cell cycling. This study is the first report to show that the expression of GPR30 is associated with a specific histological subtype. Our findings may imply that the poorer prognosis of clear cell adenocarcinoma (which is related to its resistance to chemotherapy) correlates with low GPR30 expression, and might be associated with a slow cell cycle.

## Conclusions

We herein demonstrated that the co-expression of GPR30 and EGFR was associated with a poorer progression free survival in ovarian cancer patients, and that GPR30 activates the phosphorylation of Akt via the EGFR in ovarian cancer cells. These lines of evidence reinforce our speculation that GPR30 plays an important role in ovarian cancer, other than clear cell carcinoma. The small sample size is a limitation of the present study. Therefore, further examinations will be needed to fully elucidate the functions and role(s) of GPR30. However, these studies can lead to a deeper understanding of tumorigenesis and may provide improved treatments for ovarian cancer. We believe that the regulation of GPR30 may be a potentially useful new therapeutic target in ovarian cancer.

## Abbreviations

GPR 30: G protein-coupled receptor 30; ERα: Estrogen receptor α; ERβ: Estrogen receptor β; EGFR: Epidermal growth factor receptor; EREs: Estrogen-responsive elements; E2: 17-beta-estragiol; PLC: Phospholipase C; PI3K: Phosphatidylinositol-3 kinase; ERK: Extracellular signal-related kinase; BMI: Body mass index.

## Competing interests

The authors declare that they have no competing interests.

## Authors’ contributions

SF carried out the evaluation of the immunohistochemical staining, the Western blot analysis, part of the gene expression experiments, and the statistical analysis. YT participated in conception and design of the study, supplied the TMA, and drafted the manuscript. HK, MT, SY and YT participated in the design of the study and the analysis of the clinical data. TT, ST, HS, and MK supplied the TMA material and evaluated the histology of the tumor samples and the immunohistochemical staining. AT carried out the Western blot analysis and part of the gene expression experiments, and cultured the cells. YY and MO contributed methodological knowhow and participated in the design of the study. All authors read and approved the final manuscript.

## Supplementary Material

Additional file 1 **Figure S3.** 17β-estradiol induced the phosphorylation of the EGFR and Akt in GPR30 transfected cells. Click here for file

Additional file 2 **Figure S1.** G1 induced the phosphorylation of the EGFR and Akt in A2780 cells. Click here for file

Additional file 3 **Figure S2.** G1 induced the phosphorylation of the EGFR and Akt in RMG- cells. Click here for file
