# Supplementary figures and images for: GPR30 regulates the EGFR-Akt cascade and predicts lower survival in patients with ovarian cancer
Source: J Ovarian Res. 2012 Nov 19;5:35. doi: 10.1186/1757-2215-5-35 (PMC3543193; doi:10.1186/1757-2215-5-35)

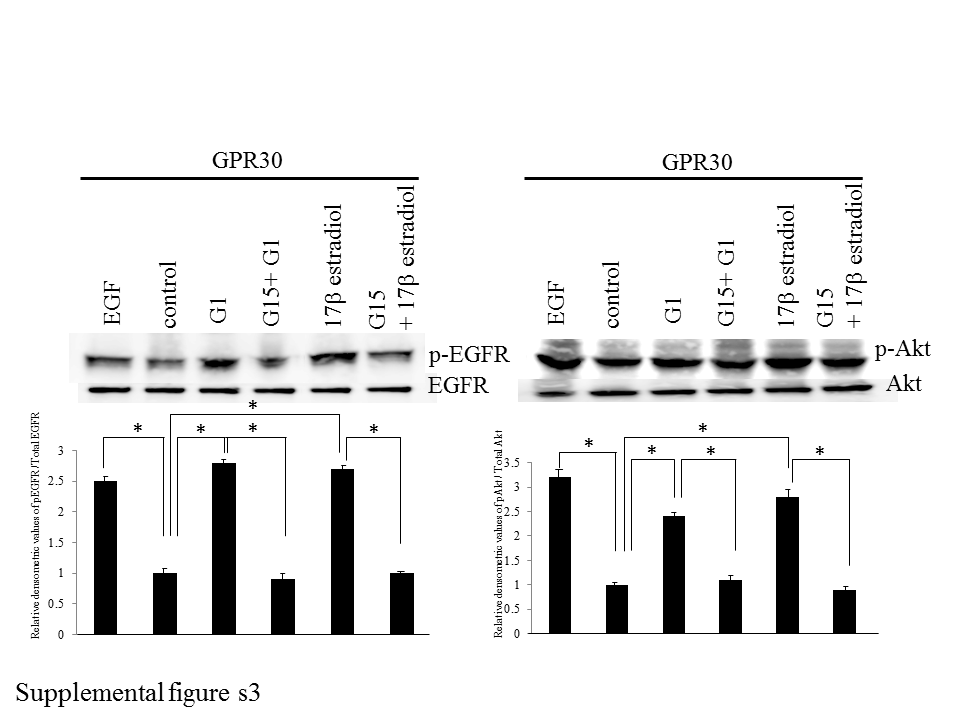

Supplement: Additional file 1 — Figure S3. 17β-estradiol induced the phosphorylation of the EGFR and Akt in GPR30 transfected cells. [file 1757-2215-5-35-S1.tiff]

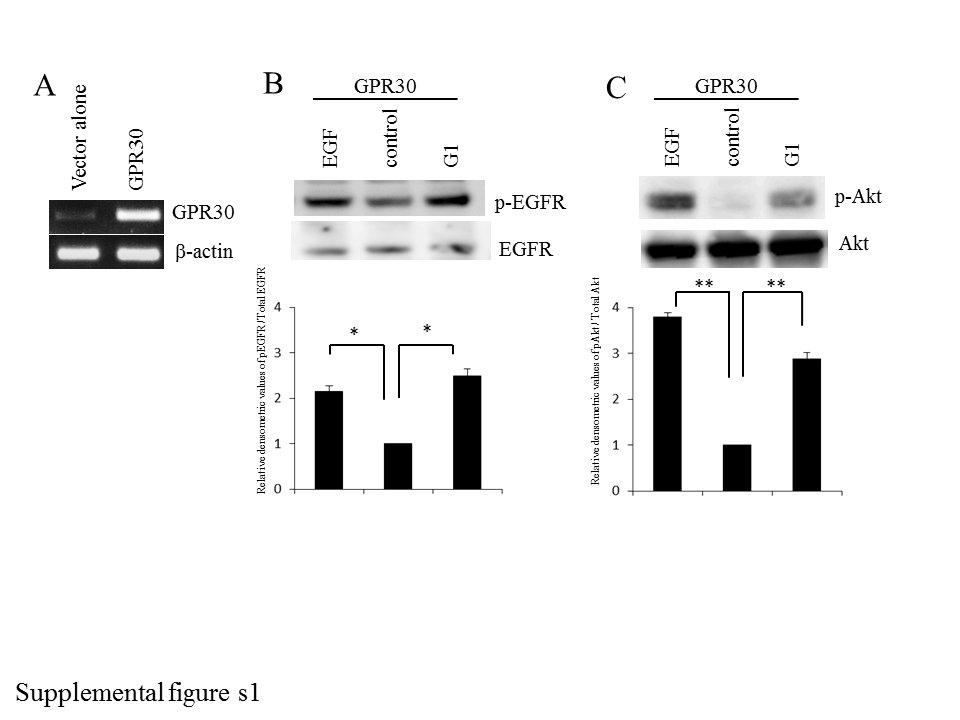

Supplement: Additional file 2 — Figure S1. G1 induced the phosphorylation of the EGFR and Akt in A2780 cells. [file 1757-2215-5-35-S2.tiff]

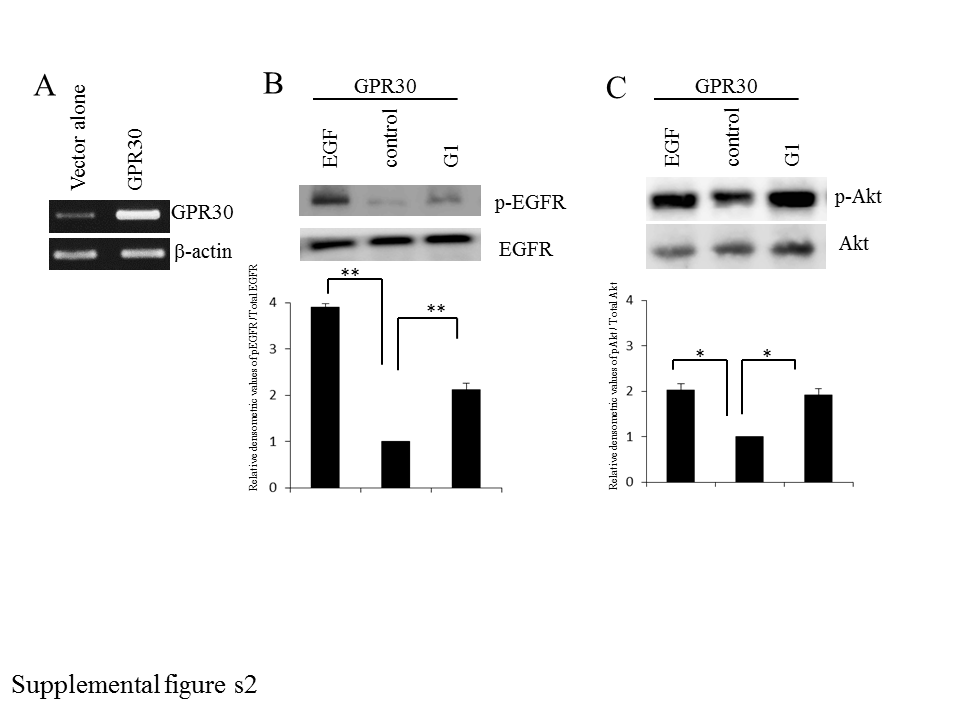

Supplement: Additional file 3 — Figure S2. G1 induced the phosphorylation of the EGFR and Akt in RMG- cells. [file 1757-2215-5-35-S3.tiff]
